# To Combine or Not to Combine Physical Therapy With tDCS for Stroke With Shoulder Pain? Analysis From a Combination Randomized Clinical Trial for Rehabilitation of Painful Shoulder in Stroke

**DOI:** 10.3389/fpain.2021.696547

**Published:** 2021-07-01

**Authors:** Janaina Andressa de Souza, João Carlos Ferrari Corrêa, Anna Marduy, Letizzia Dall'Agnol, Maria Helena Gomes de Sousa, Victor Nunes da Silva, André Barreto Alves, Soraia Micaela Silva, Felipe Fregni, Fernanda Ishida Corrêa

**Affiliations:** ^1^Doctorate and Masters Program in Rehabilitation Science of University Nove de Julho, UNINOVE, São Paulo, Brazil; ^2^Department of Physical Medicine and Rehabilitation, Neuromodulation Center, Harvard Medical School, Spaulding Rehabilitation Hospital, Boston, MA, United States

**Keywords:** stroke, transcranial direct current stimulation, shoulder pain, physical and rehabilitation medicine, physical therapy specialty

## Abstract

**Purpose:** Transcranial Direct Current Stimulation (tDCS) is an intervention that seems to be an ideal tool to enhance the effects of rehabilitation therapies given it facilitates generation of plasticity in the stimulated brain area. In stroke this strategy has been highly utilized; however, the results have been mixed. In this trial we have evaluated the analgesic and functional effects of Transcranial Direct Current Stimulation (tDCS) combined with physiotherapy in stroke survivors with shoulder pain.

**Methods:** Twenty-six stroke surviving adults with shoulder pain received 10 sessions of passive mobilization and performed upper limb exercises using a cycle ergometer, combined with active or sham tDCS. The intensity of pain in the hemiplegic shoulder was measured using the Visual Analog Scale (VAS); secondary outcomes were the level of motor impairment, handgrip strength, range of motion, motor function of the upper limbs, and quality of life (QOL) assessed before and after 10 sessions and 1 month after the end of the treatment.

**Results:** A clinically important pain reduction (3 points) was found in both groups and was maintained at follow-up; there was no significant difference between groups (*p* = 0.3). Similarly, the shoulder range of motion improved, motor function and quality of life improved showed no significant differences between groups. One result that needs to be underscored is that both groups had a significant effect size toward improvement in all of these outcomes.

**Conclusions:** We discuss in this study that tDCS is not a useful combination strategy when the physical therapy has a large effect by itself and we also review other negative trials of combined therapy under this framework of ceiling effect of the main physical therapy.

**Trial registry:** Trial registration: Brazilian Registry of Clinical Trials, RBR-8F5MNY (http://www.ensaiosclinicos.gov.br/rg/RBR-8f5mny/). Registered on June 2, 2017.

Beginning of the recruitment of the volunteers: august, 2017.

## Introduction

Transcranial direct current stimulation (tDCS) is a non-invasive, low-cost therapy that has been widely used in the rehabilitation field. It is a practical, user-friendly intervention, with minimal, mild side effects that contributes to cortical function modulation through the enhancement of neuroplasticity (1, 2).

TDCS modulates neuronal thresholds by increasing the likelihood of depolarization or hyperpolarization without inducing action potentials and facilitating spontaneous and intentional neuronal activity (3). For this reason, combining tDCS with other therapies, such as exercise, in conditions such as stroke, is thought to convey a synergistic effect; physical rehabilitation techniques induce cortical activation by inducing action potentials (4). Thus, over the years, tDCS use has been widely investigated as an adjuvant therapy to aid in the rehabilitation therapy of conditions such as fibromyalgia, depression, low back pain, and stroke (2).

Stroke rehabilitation encompasses several different fields including motor capacity, and chronic pain. In stroke, it is thought that there is an imbalance in cortical modulation where there is overexcitability of the unaffected hemisphere's cortex and a decrease in excitability in the affected hemisphere's cortex (2). Several studies have found that tDCS enhances motor function in post stroke patients either by means of ipsilesional hemisphere activation, through anodal tDCS, or inhibition of the contralesional hemisphere, through cathodal tDCS (5). In addition to motor function, the use of tDCS has been widely studied for patients with acute, subacute, and chronic stroke to approach improvement in motor function and pain (1).

Shoulder pain is a frequent consequence of stroke that leads to reduced function of the upper limb and limited performance in activities of the individual's daily life. The prevalence of shoulder pain among stroke survivors varies from 30 to 65% (6). This condition is more common from the 2nd month after stroke on and may disappear spontaneously in some cases or persist in 65% of the patients, for 12 months or more (7, 8).

Chronic pain results from permanent and inadequate changes in the sensorimotor areas of the brain, such as the primary motor cortex, the extent of the reorganization of the motor cortex being proportional to the intensity of chronic pain (7–10). The modulation of the motor cortex likely activates structures such as the thalamus, responsible for transporting information about pain to the cortex. This sensation is processed in the thalamus and cortex through the action of encephalins and β-endorphins, which have an analgesic effect and influence the perception of nociceptive stimuli due to its effect on opioid receptors (11).

In the context of post-stroke pain, tDCS has been studied as an adjuvant therapy for physical therapy techniques to enhance its beneficial effects in pain reduction. However, although tDCS as an adjuvant therapy has been proven to foster the effects of mainstay rehabilitation techniques, studies evaluating this combined therapy convey mixed results (4, 12). For instance, Bolognini et al. (13) showed that tDCS combined with constraint induced movement therapy improves motor function. On the other hand, Edwards et al. (14) have investigated the combination of robot-assisted therapy and tDCS for clinical improvement in stroke and found that tDCS does not add a synergistic effect to robot-assisted physical therapy. Accordingly, the use of tDCS as an ancillary technique in the context of post-stroke pain prompts further investigation.

Considering the relationship between pain and reduced motor function and the fact that long periods of cortical stimulation can have lasting effects on cerebral function (15, 16), we hypothesize that combining tDCS and physiotherapy could lead to a reduction in shoulder pain among stroke survivors, with a consequent improvement in functioning and quality of life. Therefore, the primary aim of the present study was to determine whether tDCS can enhance the effects of physiotherapy on shoulder pain in stroke survivors. The secondary objective was to correlate the analgesic effects of treatment with motor function, motor impairment, and quality of life.

## Methods

The study is in accordance with the principles of the Declaration of Helsinki and with the norms and guidelines for research on human beings, formulated by the National Health Council of Brazil, created in October 1996. It received approval from the Human Research Ethics Committee by Nove de Julho University, São Paulo, Brazil (number 2.038.903), and was registered in the Brazilian Registry of Clinical Trials (RBR-8F5MNY).

A longitudinal, randomized, controlled and double-blind clinical trial was carried out, in which 26 stroke, aged >18 years, victims with chronic shoulder pain (minimum of 6 months) were selected according to the International Association for the Study of Pain (IASP) (17).

To be included in the study, subjects should be able to understand commands (as indexed by a Mini-Mental State Exam score >11) (18). Individuals were excluded if they have:

- a contraindication for non-invasive brain stimulation, such as metallic implants close to the application sites,
- a history of seizures and/or epilepsy, pregnancy, and diagnosis of neoplasia (19),
- muscular inelasticity (spasticity) >3 in the affected upper limb, assessed by the Modified Ashworth Scale (20);
- progressive neurological disease;
- frozen shoulder diagnosis;
- severe sensory deficit (score >2) on the National Institutes of Health (NIH) Stroke Scale (21);
- a diagnosis of acute coronary syndrome or severe heart problem (score >3 on the New York Heart Association functional rating scale) (22);
- severe aphasia [score >2 (assessed in the patient's native language) indicated by the NIH Stroke Scale] (21);
- suspected or confirmed recent upper limb fracture;
- cancer diagnosis and/or palliative care therapy; and
- severe inattention (score >2 indicated by the NIH Stroke Scale) study (21).

Concomitant care and interventions prohibited during the trial included: the recent use of botulinum toxin or injection of phenol in the affected upper limb (previous 3 months) or a medical indication for use during the study period; use of medications that could affect assessments (anti-inflammatory steroids) and participation in physical or homeopathic therapy during the study.

According to the International Association for the Study of Pain (IASP) (17), three consecutive months of complaints of pain are considered the cutoff point between acute and chronic pain, however, for research purposes, chronic pain is considered after six consecutive months.

The elected participants were randomized into two groups using opaque envelopes, by a researcher not involved in evaluations or treatment: (1) active tDCS combined with physiotherapy for the upper limbs; (2) sham tDCS combined with physiotherapy for the upper limbs.

### Procedures

**Figure 1:**
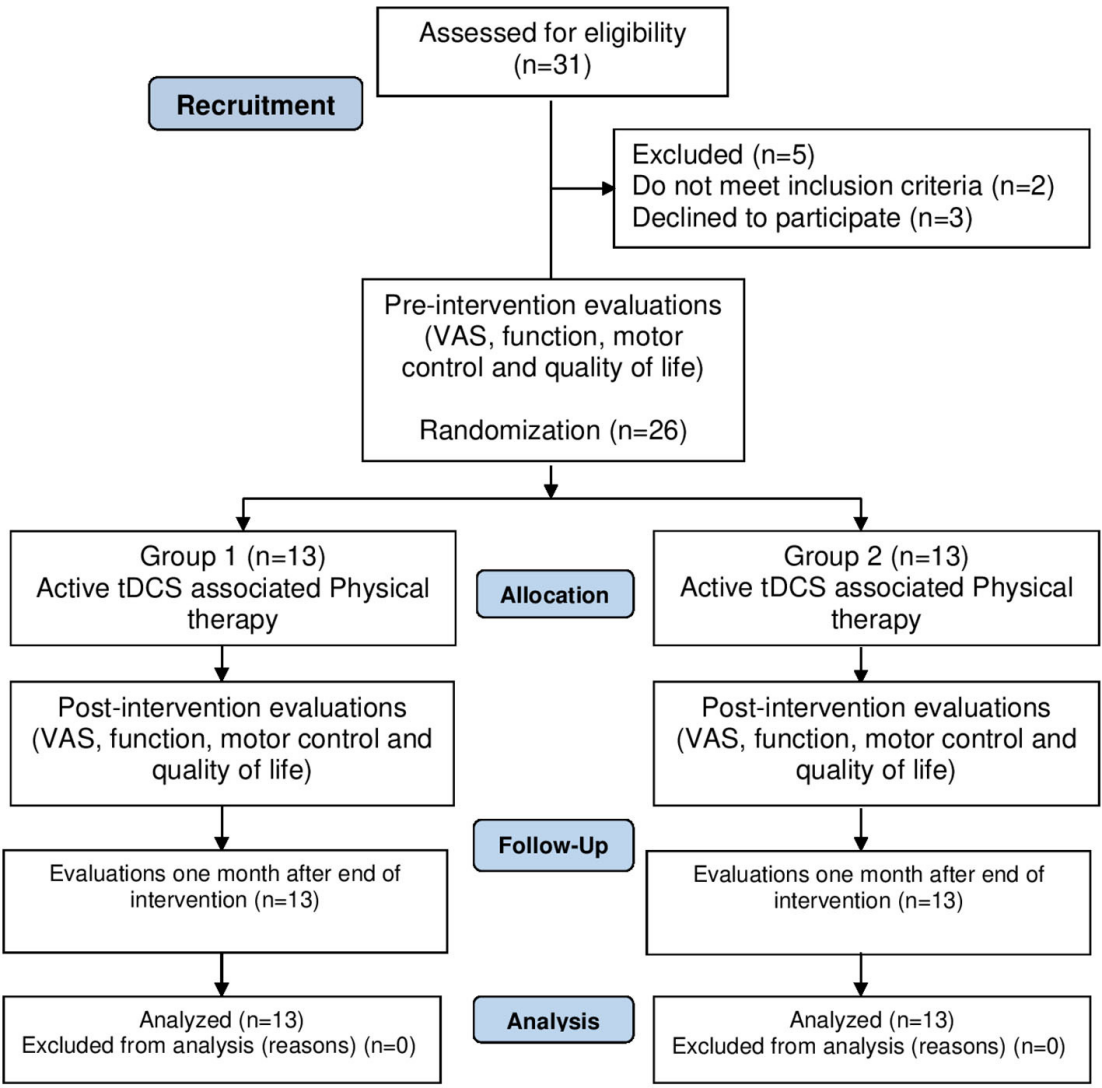
Consort diagram illustrating the process from recruitment to data collection.

Evaluations were performed on three occasions: pre- and post-intervention and follow-up (30 days after completion of the intervention). The details of the study procedure are shown in Figure 1 and can be seen in the protocol article published by our group (23).

The primary outcome was shoulder pain intensity measured using the visual analog scale (VAS) (24). Evaluations were performed with the patient at rest, as well as during flexion, abduction, and external rotation active movement (performed by participant) and flexion, abduction, and external rotation passive movement (performed by a researcher qualified to perform the movement and who was also blind to the allocation of patients).

The secondary outcomes were the dysfunction and disability associated with shoulder pain measured by the Shoulder Pain and Disability Index (SPADI) (25); symptoms and disabilities caused by upper limb disorders were assessed using the Disabilities of the Arm, Shoulder and Hand (DASH) questionnaire (26); upper limb motor impairment was analyzed using the Fugl-Meyer Assessment of Motor Recovery after Stroke Scale (27, 28) (for the present study only the upper limbs score was used); the range of motion of the shoulder (RMS) for external rotation, abduction and flexion was measured using a goniometer (29); grip strength was measured in both hands using a dynamometer (Jamar®, Enterprises Inc., Irvington, New York, USA) (30), and the *Quality of life* was measured using the Stroke Specific Quality of Life Scale (SSQOL) (31).

The confounding factors were evaluated by the depressive symptoms using the Beck Depression Inventory (BDI) (32, 33), and by sleep disorders using the Pittsburgh Sleep Quality Index (PSQI) (34).

### Treatment Physiotherapy

The treatment was carried out 5 days a week for two consecutive weeks, totaling 10 sessions (35, 36). Each session involved 20 min of passive exercises and 20 mi of active exercises for upper limbs combined with active-anodal tDCS or sham tDCS.

The physiotherapeutic treatment was always performed by the same therapist and passive therapy consisted of massage, stretching, and joint mobilization with the patient in the supine position, for 20 min (23). Then the participant received the tDCS combined with active or active-assisted exercises, using a cycle ergometric (Acte Sports, model E5) for another 20 min. The cycle ergometer was positioned on a table, in front of the patient (23). The patient was seated to perform the exercise.

### tDCS Treatment

Transcranial direct current stimulation was performed using the “NeuroConn DC-STIMULATOR PLUS” administered over the primary motor cortex. The anode (5 × 5 cm) was positioned over the damaged hemisphere (C3 or C4) according to the 10/20 international electroencephalogram system (19) and the cathode (5 × 7 cm) was positioned in the supraorbital region contralateral to the anode (19, 35, 37, 38). Both electrodes were enveloped in sponge soaked in saline solution, 2 mA, 30 s of ramp up and ramp down. The sham tDCS was performed with the same assembly as the active therapy, but the current was administered only for 60 s to give the participant the sensation of stimulation.

The tDCS—Side Effects Questionnaire (39) was administered after each session to determine the occurrence of possible side effects stemming from non-invasive brain stimulation.

### Blinding

Blinding of the participants and therapist was achieved with the sham mode of the tDCS device. The device was programmed by another researcher. Thus, neither the therapist nor the patient was aware of which mode (active or sham) was selected, as the external functioning of the device is the same in both modes.

### Statistical Analysis

Data analysis involved intention-to-treat analysis. The Shapiro-Wilk test was used to determine the normality of the data. The data were parametric and were therefore expressed as mean and standard deviation values. Two-way analysis of variance (ANOVA) followed by the Bonferroni test was used for the inter-group comparisons. Pearson's correlation coefficients were calculated to determine the strength of correlations between pain and the secondary outcomes. A regression analysis was performed to determine the effect of possible confounding factors. All analyses were processed using the SPSS program (IBM SPSS Statistics for Windows, version 22.0, released in 2013, IBM Corp Armonk, NY, USA) and a *p* < 0.05 was considered indicative of statistical significance.

## Results

Thirty-one patients were screened and five were excluded (three declined to participate, one had shoulder luxation and one did not feel continuous pain for a period of 6 months). Thus, the sample was composed of 26 patients. Table 1 displays the demographic data of the participants.

**Table 1:** Characteristic of sample at baseline (*n* = 26).

**Characteristics**	**Active tDCS combined with physical therapy (*n* = 13)**	**Sham tDCS combined with physical therapy (*n* = 13)**
Sex (F/M)	13 (4/9)	13 (5/8)
Age (years)	54.6 ± 10.3	54.3 ± 10.5
**VAS (score)**		
Rest	5.4 ± 1.6	4.1 ± 1.0
Active	4.0 ± 2.4	3.9 ± 1.2
Passive	4.6 ± 2.4	4.3 ± 1.7
**Type of stroke**		
Ischemic/hemorrhagic	12/1	11/2
**Hemiparesis**		
Right/left	7/6	7/6
Duration of pain (months)	29.3 ± 19.4^*^	35.0 ± 22.8^*^
Time elapsed since stroke (months)	35.6 ± 21.9^*^	41.6 ± 23.6^*^
Fugl-Meyer (upper limbs)	21.9 ± 18.7	41.5 ± 27.2^*^
**ROM of shoulder**		
Flexion	131.2 ± 32.4	144.9 ± 33.4
Abduction	113.0 ± 34.4	126.6 ± 34.5
External rotation	44.3 ± 18.6	50.0 ± 23.1
Grip strength	5.9 ± 7.0	12.6 ± 6.8
**Motor function**		
SPADI	84.7 ± 8.6	71.2 ± 16.1
DASH	61.8 ± 16.6	45.2 ± 19.5
SSQOL	145.5 ± 26.7	167.7 ± 26.7
BDI	18.3 ± 14.7	13.5 ± 9.6
Pittsburg	7.6 ± 4.6	7.3 ± 4.2
Use of antidepressants	04 (30.7%)	02 (15.3%)
Use of acetylsalicylic acid	09 (69.2%)	09 (69.2%)
Use of diuretics	03 (23.0%)	06 (46.1%)
Use of anti-hypertensives	11 (84.6%)	11 (84.6%)
Use of medication for diabetes	01 (7.6%)	03 (23.0%)
Use of medication for cholesterol	11 (84.6%)	10 (76.9%)
Use of medication for anxiety	01 (7.6%)	02 (15.3%)
**Associated comorbidities**		
Hypertension	11 (84.6%)	11 (84.6%)
Diabetes	01 (7.6%)	03 (23.0%)
Dyslipidemia	11 (84.6%)	10 (76.9%)

*Data expressed as mean ± standard deviation and frequency (%); F/M, female/male; VAS, visual analog scale; tDCS, transcranial direct current stimulation; ROM, range of motion; SPADI, Shoulder Pain and Disability Index; DASH, Disabilities of the Arm, Shoulder and Hand; SSQOL, Stroke Specific Quality of Life Scale; BDI, Beck Depression Inventory; Pittsburg, Pittsburgh Sleep Quality Index*.

* *P < 0005 (Anova two-way)*.

The two groups were similar in terms of pain intensity at rest and during active and passive movements, sex, age, type of stroke. Significant differences were found between the two groups in terms of pain duration and time since stroke (*p* < 0.05-Anova two-way), with pain and injury time being greater for the sham group. The pain time for the sham group was 35.0 ± 22.8 and injury time 41.6 ± 23.6 and for the active group the pain time was 29.3 ± 19.4 and injury time 35.6 ± 21.9. Greater impairment of the upper limb was also observed, assessed by the Fugl-Meyer scale, for the sham group (*p* < 0.05) with an average of 41.5 ± 27.2 when compared to the active group 21.9 ± 18.7.

Most individuals in both groups used antihypertensive drugs, acetylsalicylic acid, and cholesterol-lowering drugs. Two individuals (one in each group) took an analgesic only once during the study due to unbearable pain (orphenadrine citrate, metamizole sodium monohydrate, anhydrous caffeine, paracetamol). All had at least one associated comorbidity, the most prevalent being hypertension and dyslipidemia.

Table 2 displays the pain levels at rest as well as during active and passive movement measured using the VAS at the pre-intervention, post-intervention and 30-day follow-up evaluations in the two groups.

Table 2Pain intensity (VAS) at different evaluation times in groups submitted to active and sham tDCS combined with physical therapy (intra-group and inter-group differences).

**Table:** 

**Variables (VAS)**	**Active tDCS and physical therapy**			**Sham tDCS and physical therapy**			**Inter-group difference**
	**Pre-treatment**	**Post-treat**	**30-day follow-up**	**Pre-treatment**	**Post-treat**	**30-day follow-up**	**MD (95% CI)**
Pain -rest	5.4 ± 1.6^†^^,^^††^	0.4 ± 0.6^†^	0.5 ± 0.9^††^	4.1 ± 1.0^†^^,^^††^	0.4 ± 0.6^†^	0.7 ± 1.1^††^	0.33 (−0.31 to 0.98)
Pain—passive	4.6 ± 2.4^†^^,^^††^	2.1 ± 2.1^†^	1.9 ± 2.0^††^	4.3 ± 1.7^†^^,^^††^	1.2 ± 1.7^†^	1.6 ± 1.9^††^	0.51 (−0.90 to 1.93)
Pain—active	4.0 ± 2.4^†^^,^^††^	1.0 ± 1.6^†^	0.9 ± 1.3^††^	3.9 ± 1.2^†^^,^^††^	1.0 ± 1.4^†^	1.1 ± 1.4^††^	0.00 (−1.06 to 1.06)

**Table:** 

	**Intra-group difference**		**Intra-group difference**	
	**Post** ***vs***. **pre MD (95% CI)**	**Follow-up** ***vs***. **pre D (95% CI)**	**Post** ***vs***. **pre MD (95% CI)**	**Follow-up** ***vs***. **pre MD (95% CI)**
Pain—rest	−5.0 (−6.0 to −3.9)	−4.8 (−5.8 to −3.8)	−3.87 (−4.7 to −2.7)	−3.4 (−4.4 to −2.3)
Pain—passive	−2.53 (−3.69 to −1.38)	−2.69 (−4.18 to −1.19)	−3.03 (−4.19 to −1.88)	−2.69 (−4.18 to −1.19)
Pain—active	−2.92 (−4.33 to −1.51)	−3.03 (−4.37 to −1.69)	−2.92 (−4.33 to −1.51)	−2.80 (−4.1 to −1.46)

*Data expressed as mean ± standard deviation;*

†, *difference between post and pre (p ≤ 0.05)*;

††, *difference between follow-up and pre (p ≤ 0.05) (Anova two-way); VAS, visual analog scale; MD, mean difference; CI, confidence interval*.

A clinically important reduction in pain (3 points on the vas) was found in both groups. the best results were found with the patients at rest (vs. passive and active movement). However, no significant inter-group difference was found for pain at rest (*p* = 0.3) or during active (*p* = 1.0) or passive (*p* = 0.46) movement. The effect size (measured using cohen's d) was large for both groups (*d* = 0.89 at post-intervention evaluation) and (*d* = 0.87 at follow-up evaluation in active tdcs group); (*d* = 0.90 at post-intervention evaluation) and (*d* = 0.84 at follow-up evaluation in sham group).

Table 3 displays the grip strength and range of motion values at the pre-intervention, post-intervention and 30-day follow-up evaluations in both groups.

Table 3Grip strength and shoulder range of motion at different evaluation times in groups submitted to active and sham tDCS combined with physical therapy (intra-group and inter-group differences).

**Table:** 

**Variables**	**Active tDCS and physical therapy**			**Sham tDCS and physical therapy**			**Inter-group difference**
	**Pre-treatment**	**Post-treat**.	**30-day follow-up**	**Pre-treatment**	**Post-treat**.	**30-day follow-up**	**MD (95% CI)**
Grip strength (KgF)	5.9 ± 7.0	8.3 ± 7.8	8.0 ± 8.3	12.6 ± 6.8	13.4 ± 7.9	14.3 ± 8.4	–
ROM—flexion (degrees)	131.2 ± 32.4^†^^,^^††^	155.9 ± 30.3^†^	154.4 ± 30.9^††^	144.9 ± 33.4	153.2 ± 31.0	147.3 ± 32.0	−1.2 (−26.2 to 23.6)
ROM—abduction (degrees)	113.0 ± 34.4^†^^,^^††^	143.5 ± 37.3^†^	141.3 ± 33.9^††^	126.6 ± 34.5	140.2 ± 44.4	136.3 ± 38.2	−1.7 (−29.5 to 26.0)
ROM—ext. rot. (degrees)	44.3 ± 18.6^†^	56.8 ± 22.0^†^	53.0 ± 19.2	50.0 ± 23.1	53.3 ± 21.2	56.4 ± 23.7	−1.8 (−18.0 to 14.3)

**Table:** 

	**Intra-group difference**		**Intra-group difference**	
	**Post** ***vs***. **pre MD (95% CI)**	**Follow-up** ***vs***. **pre MD (95% CI)**	**Post** ***vs***. **pre MD (95% CI)**	**Follow-up** ***vs***. **pre MD (95% CI)**
Grip strength (KgF)	2.3 (−0.3 to 5.0)	2.0 (−0.9 to 5.0)	0.7 (−1.9 to 3.4)	1.6 (−1.3 to 4.6)
ROM—flexion (degrees)	24.6 (15.7–33.6)	23.2 (12.2–34.1)	8.3 (−0.6 to 17.2)	2.3 (−8.5 to 13.30)
ROM—abduction (degrees)	30.4 (9.4–51.4)	28.3 (9.2–47.3)	13.5 (−7.4 to 34.5)	9.6 (−9.6 to 28.7)
ROM—ext. rot. (degrees)	12.4 (2.0–22.8)	8.6 (−0.02 to 17.4)	3.3 (−7.0 to 13.8)	6.4 (−2.2 to 15.1)

*Data expressed as mean ± standard deviation;*

†, *difference between post and pre (p ≤ 0.05)*;

††, *difference between follow-up and pre (p ≤ 0.05) (Anova two-way); MD, mean difference; CI, confidence interval; ROM, range of motion*.

No improvements in grip strength were found after the intervention in either group. Regarding ROM (flexion, abduction and external rotation), a statistically significant (*p* < 0.05) increase was found in the active tDCS at the post-intervention and follow-up evaluations compared to the pre-intervention evaluation. However, no significant inter-groups differences were found (*p* > 0.05).

Table 4 displays the results of the motor function, motor performance and quality of life assessments at the pre-intervention, post-intervention and 30-day follow-up evaluations in both groups.

Table 4Motor function, motor impairment and quality of life at different evaluation times in groups submitted to active and sham tDCS combined with physical therapy (intra-group differences).

**Table:** 

**Variables**	**Active tDCS and physical therapy**			**Sham tDCS and physical therapy**		
	**Pre-treatment**	**Post-treat**.	**30-day follow-up**	**Pre-treatment**	**Post-treat**.	**30-day follow-up**
DASH (0–100)	61.8 ± 16.6^††^	56.3 ± 16.2	49.3 ± 13.8^††^	45.2 ± 19.5^†^^,^^††^	33.2 ± 15.4^†^	31.7 ± 16.9^††^
SPADI (0–100)	84.7 ± 8.6^†^^,^^††^	64.3 ± 13.4^†^	60 ± 18.4^††^	71.2 ± 16.1^†^^,^^††^	56.3 ± 27.3^†^	53.4 ± 23.4^††^
Fugl-Meyer—upper limbs (0–66)	21.9 ± 18.7	25.6 ± 21.1	26.4 ± 21.0	41.5 ± 27.2	41.4 ± 27.0	40.9 ± 26.0
SSQOL (total: 245 points)	145.5 ± 26.7^†^^,^^††^	167 ± 32.8^†^	167.6 ± 30.3^††^	167.7 ± 26.7^†^^,^^††^	184 ± 19.8^†^	185.1 ± 24.7^††^

**Table:** 

	**Intra-group difference**		**Intra-group difference**	
	**Post** ***vs***. **pre MD (95% CI)**	**Follow-up** ***vs***. **pre MD (95% CI)**	**Post** ***vs***. **pre MD (95% CI)**	**Follow-up** ***vs***. **pre MD (95% CI)**
DASH (0–100)	−5.5 (−13.6 to 2.5)	−12.5 (−22.4 to −2.6)	−11.9 (−20 to −3.8)	−13.4 (−23.3 to −3.5)
SPADI (0–100)	−20.3 (−33.7 to −7.0)	−24.6 (−38.3 to −10.8)	−14.9 (−28.2 to −1.5)	−17.8 (−31.5 to −4.0)
Fugl–Meye—upper limbs (0–66)	3.6 (−0.1 to 7.5)	4.5 (−0.8 to 9.9)	−0.07 (−3.9 to 3.7)	−0.6 (−5.9 to 4.7)
SSQOL (total: 245 points)	21.4 (8.2–34.7)	22 (9.1–35)	17 (3.8–30.3)	17.3 (4.4–30.3)

*Data expressed as mean ± standard deviation;*

†, *difference between post and pre (p ≤ 0.05)*;

††, *difference between follow-up and pre (p ≤ 0.05) (Anova two-way); MD, mean difference; CI, confidence interval; DASH, Disabilities of the Arm, Shoulder and Hand (0–100 points, higher scores indicative of worse condition); SPADI, Shoulder Pain and Disability Index (0–100 points, higher scores indicative of worse condition); SSQOL, Stroke Specific Quality of Life Scale (245 points, higher scores indicative of better quality of life); Fugl-Meyer (0–100 points, 34 for lower limbs and 66 for upper limbs)*.

No improvements were found in motor impairment (Fugl-Meyer scale) in any of the groups. On the other hand, motor function (DASH scale) improved in both groups, post-intervention in the active tDCS group and post-intervention and follow-up in the sham group. Regarding the results of SPADI, post-intervention and follow-up improvements were found for both groups. Quality of life (SSQOL) improved post-intervention and was maintained at follow-up for both groups.

No intergroup analyzes were performed for motor function, motor impairment or quality of life due to significant differences between the pre-intervention groups (DASH: *p* = 0.028; SPADI: *p* = 0.014; Fugl-Meyer: *p* = 0.043; SSQOL: *p* = 0.045).

### Measurement of Potential Side Effects

It was reported in 40% of the individuals itching sensation on the scalp, 20% headache, 53% tingling sensation, 46% burning sensation, 20% drowsiness, and 20% neck pain. Most individuals reported a burning, itchy, and tingling sensation at the beginning of each stimulation, and headache, neck pain, and drowsiness were rarely reported at the end of the sessions.

Table 5 displays the results of the regression analysis for the adjustment of confounding variables.

**Table 5:** Modeling for adjustment of confounding variables of associations between pain and age, time since stroke, duration of pain, BDI, Pittsburg, and Fugl-Meyer.

	**Unadjusted -coefficient**	**Standard error**	**Adjusted -coefficient**	**95% CI**	***P*-value**
**Active-tDCS model**					
Active tDCS	−10.313	4.134	–	−20.430 to −0.197	0.047
Age	0.052	0.065	0.288	−0.108 to 0.213	0.454
Time since stroke	0.062	0.130	0.717	−0.256 to 0.379	0.652
Time of ongoing pain	−0.019	0.143	−0.196	−0.369 to 0.331	0.899
BDI	0.008	0.078	0.060	−0.184 to 0.199	0.925
Pittsburg	−0.022	0.265	−0.053	−0.671 to 0.627	0.937
Fugl–Meyer Upper Limb	0.038	0.036	0.379	−0.051 to 0.127	0.337
**Sham-tDCS model**					
Sham tDCS	−3.861	1.549	–	−7.650 to −0.071	0.047
Age	−0.022	0.031	−0.332	−0.097 to 0.053	0.503
Time since stroke	0.002	0.055	0.057	−0.132 to 0.135	0.977
Time of ongoing pain	0.005	0.058	0.162	−0.138 to 0.148	0.935
BDI	0.037	0.042	0.516	−0.066 to 0.140	0.516
Pittsburg	0.011	0.090	0.066	−0.208 to 0.230	0.066
Fugl-Meyer Upper Limb	0.012	0.014	0.466	−0.023 to 0.047	0.438

*Dependent variable, Delta of pain at rest; β, beta coefficient; BDI, Beck Depression Inventory; Pittsburg, Pittsburgh Sleep Quality Index; Fugl-Meyer Upper Limb, Fugl-Meyer scale for upper limbs. Model regression analysis*.

The confounding variables did not exert an influence on the pain outcome. Moreover, Pearson's correlation coefficients revealed that pain was not correlated with any of the secondary outcomes in the active tDCS group (DASH [*p* = 0.3], SPADI [*p* = 0.2], Fugl-Meyer [*p* = 0.1], and SSQOL [*p* = 0.3]) or sham group (DASH [*p* = 0.9], SPADI [*p* = 0.8], Fugl-Meyer [*p* = 0.6], and SSQOL [*p* = 0.8]).

## Discussion

A clinically important reduction in pain at rest (3 points on VAS) (24) was found in both groups. Concomitantly, large effect sizes in the post-intervention assessment, as well as in the follow-up assessment in the active anodic tDCS and sham tDCS groups, respectively were observed. Pain during passive and active movement was also reduced in both groups. These results allow us to infer that the addition of anodic tDCS over the injured primary motor cortex did not increase the analgesic effect of physical therapy in stroke survivors with shoulder pain.

A similar result was also observed in a study by Belley et al. (40), in which they evaluated the effects of adding active tDCS (a-tDCS) and sham tDCS during a rehabilitation program focused on sensorimotor training in individuals with rotator cuff tendinopathy. The results did not demonstrate any improvement in pain in the treatment with the addition of a-tDCS during a rehabilitation program for these individuals, finding level 1b of Evidence of Therapy. The convergence between the present results and the findings reported by other authors may be related to the ceiling effect of tDCS.

### Ceiling Effect of Non-invasive Brain Stimulation

The results presented in our study with a clinically important reduction in VAS pain in both groups reinforce the notion that the combination of tDCS and physical therapy for post-stroke shoulder pain created a ceiling effect in which the benefits of physical therapy overshadowed those of tDCS (41). This is a common pattern in stroke rehabilitation, as several studies have conveyed this effect (14, 42).

Edwards et al. (14) evaluated the effectiveness of robot-assisted therapy with tDCS compared to sham tDCS for clinical improvement of post-stroke patients. Robot-assisted therapy was shown to be effective for clinical improvement in both groups, with no significant differences between them. In fact, both tDCS and sham tDCS groups had large improvements in motor function (6.97 and 7.73 in Fugl-Meyer score, respectively). Surely, in this case, the goal of neuromodulation to enhance the effects of physical therapy becomes null as therapy alone was able to maximize its effects.

Similarly, Harvey et al. (42) assessed the use of goal-oriented arm and hand therapy with resting transcranial magnetic stimulation (rTMS) in stroke patients. Once again, in the sham and active groups, significant motor function improvement was conveyed. Similarly, to Edwards et al. (14), results, both groups had a large improvement. Sixty-seven percent of the active TDCS group and 65% of sham TDCS group had more than 5 point improvement in their Fugl-Meyer scores. In this protocol, goal-directed hand and arm tasks facilitated outcome achievement in both groups, leading to a ceiling effect when combining rTMS with these tasks (42).

This, however, does not suggest that tDCS and other neuromodulation techniques are not effective for the improvement of stroke rehabilitation as other studies conveyed effective results in function and pain improvement when tDCS and other brain stimulation techniques are used alone. One important strategy in the future will be to compare tDCS against physical therapy when physical therapy can induce larger effects and then leave the combined strategy to the low or no responders to physical therapy.

In fact, trials with a different design showed the effects of tDCS to be effective. Bae et al. (36) found improvement in post-stroke pain in the group that received active tDCS, but not in the group submitted to tDCS sham. Antal et al. (43) found significant effects of active tDCS for trigeminal nerve neuralgia, post-stroke pain syndrome, back pain, and fibromyalgia, and Thibaut et al. (44) for pain after spinal cord injury. However, the authors did not combine stimulation with physiotherapy or other therapies. Only a study by Straudi et al. (4) for chronic back pain combined tDCS with an exercise program; the other studies used tDCS only to reduce pain. However, in the study of Straudi et al. (4) pain improvements were much more modest, suggesting this group was likely more refractory, thus being a situation in which combination provided a better outcome.

### Mechanisms of Pain Improvement

The participants in the present study had shoulder pain due to a stroke for more than six consecutive months, which caused discomfort and difficulty in performing simple tasks of daily living. Patients reported that such functional limitations were due to motor impairment, which harmed the quality of life.

A behavior change was observed after the start of treatment, as the participants from both groups were able to be more communicative and happier. All data obtained with the treatment and none were missing from any of the preparations. The participants reported a gradual reduction in pain, absent or almost absent at the end of treatment, depending on the type of activity they performed. An improvement in the perception of muscle tone of the affected shoulder has also been reported (“now my shoulder and muscles are looser”). These changes were still evidence and the pain continued to subside 30 days after the end of treatment.

Improvements without shoulder range of motion (abduction, flexion, and external rotation) are believed to have occurred due to physical therapy. Joint mobilization was performed on the affected shoulder, along with massage and along with all the muscles of the upper limbs, which probably contributed to the release of the hypertonic muscles (flexors, adductors, and internal rotators of the shoulder).

Although it is recognized that even for chronic pain (CP) triggered directly by peripheral structures, such as joint and muscle, there is a wide range of changes in the central nervous system (CNS) (45, 46). These alterations lead to a series of central changes that allow the perpetuation and maintenance of chronic pain state (45, 46). Thus, it is observed that pain is linked to maladaptive plasticity in the CNS (47–50).

Therefore, the analgesic effect of motor cortex stimulation for these individuals may occur because this area is correlated with motor-related cortical and top-down cortico-subcortical changes. For instance, there a likely top-down modulation of the descending inhibitory pain control induced by physical therapy motor activation, leading also to the release of endogenous opioids in the anterior cingulate cortex, anterior insula, and periaqueductal gray (PAG) (51). In healthy rats without neuropathic conditions, stimulation of the motor cortex reduces the responsiveness of spinal nociceptive neurons, thereby increasing the nociceptive threshold through endogenous opioids (52, 53). In neuropathic rats, stimulation of the motor cortex reverses central and peripheral pain by activating the limbic system and PAG and inhibiting the thalamic nuclei and spinal nociceptive neurons (54). Thus, it has been hypothesized that, in humans and animals, stimulation of the motor cortex induces analgesia by activating the descending analgesic pathways and neurocircuits involved in the emotional component of pain (55, 56). However, it is not clear which nuclei of the midbrain are modulated after cortical stimulation or how they act on spinal nociceptive neurons to raise the nociceptive threshold.

### Placebo Effects and Physical Therapy

Another explanation for the pain reduction results surpassing any expected effect size for the combination of physical therapy and tDCS may be a placebo effect in the sham group. While physical therapy alone can facilitate motor cortical excitability, a 3-point reduction in shoulder pain could be explained by a biologically assisted effect, caused by a placebo influence. Moreover, the thought of receiving combined therapy could have also influenced subjects in both groups to put more effort in physical therapy and thus better perform, increasing pain reduction (14, 57).

Neuromodulation techniques are also thought of influencing cortical areas that interfere with expectations. Brain networks activated by the anticipation of therapeutic benefit are modulated by tDCS, strengthening the placebo response even further (58). This modulatory activation is commonly seen in protocols evaluating the effects of tDCS in depression; the expectation of improvement promotes modulated active placebo responses, increasing the effect of tDCS therapy (59).

The possibility of the placebo effect contributing to the significant reduction in pain in the sham-tDCS group prompts performing an analysis of the combined therapy through run-in trials that can exclude high placebo responders (60). This also raises an interesting issue: clinical trials may have a greater effect in subjects' expectation and conditioning effect leading to a greater placebo effect than in clinical practice. Because of that, would effects of physical therapy be smaller in real life? If so, would then tDCS combination be attractive. This question is difficult to be answered as only observational studies may help to address this question.

Some limitations of this study must be considered, the first was the lack of analysis of the integrity of the motor cortex through electrophysiological or image evaluations, the second concerns the possible effect found in the pilot study, which may have influenced the sample size. Moreover, a significance in the duration of post-shoulder pain was found in the sham-tDCS group. This is a direct result of a small sample size and could have affected the results presented for the sham group in this study. However, regression analysis accounting for pain duration did not show significance of pain duration as a confounder in either group, conveying low likelihood that the longer pain duration in the sham group affected the trial's results. Despite these limitations, the present findings demonstrate the variability of tDCS responses to chronic pain. Showing that the effectiveness of adding tDCS to other therapies will depend on the therapy applied. In this case, it appears that the therapy alone had a good effect on shoulder pain from a stroke.

### Conclusion

Active tDCS administered over the damaged primary cortex in stroke survivors did not enhance the analgesic effect of physiotherapy in the present study. We hypothesized that the large effect of physical therapy alone and possibly combined with a placebo effect of tDCS induced a large clinical effect that prevented the potential additive effects of TDCS. We hope this discussion on the ceiling effects will help future study designs of combination therapy.

## Data Availability Statement

The raw data supporting the conclusions of this article will be made available by the authors, without undue reservation.

## Ethics Statement

The studies involving human participants were reviewed and approved by Research Ethics Committee Nove de Julho University (CoEP-UNINOVE), São Paulo, Brazil Comissão Nacional de Ética em Pesquisa (CONEP), Brazil. The patients/participants provided their written informed consent to participate in this study.

## Author Contributions

FC and JA conceived, planned the theory, and drafted the manuscript. FF and AM contributed to drafting the manuscript. JA, VN, and AA carried out the experiment. JF and FC helped supervise the project. FC and LD'A conceived the original idea. SS and MG participated the performed the statistical analysis. FC oriented the project and coordinated the project. All authors have made substantive contributions to the manuscript and assume full responsibility for its content, conceived of the study, participated in its design and coordination, and helped to draft the manuscript, read and approved the final manuscript, and agree to be accountable for the content of the work.

## Conflict of Interest

The authors declare that the research was conducted in the absence of any commercial or financial relationships that could be construed as a potential conflict of interest.

## References

[1] Zhao H Qiao L Fan D Zhang S Turel O Li Y. Modulation of brain activity with noninvasive transcranial direct current stimulation (tDCS): clinical applications and safety concerns. Front Psychol. (2017) 8:685. 10.3389/fpsyg.2017.00685 28539894 PMC5423956

[2] Fregni F Pascual-Leone A. Technology insight: noninvasive brain stimulation in neurology—perspectives on the therapeutic potential of rTMS and tDCS. Nat Clin Pract Neurol. (2007) 3:383–93. 10.1038/ncpneuro0530 17611487

[3] El-Hagrassy MM Jones F Rosa G Fregni F. CNS non-invasive brain stimulation. In: Gilleran JP, Alpert SA, editors. Adult and Pediatric Neuromodulation. Cham: Springer (2018). p. 151–84. 10.1007/978-3-319-73266-4_12

[4] Straudi S Buja S Baroni A Pavarelli C Pranovi G Fregni F. The effects of transcranial direct current stimulation (tDCS) combined with group exercise treatment in subjects with chronic low back pain: a pilot randomized control trial. Clin Rehabil. (2018) 32:1348–56. 10.1177/0269215518777881 29783893

[5] Fregni F El-Hagrassy MM Pacheco-Barrios K Carvalho S Leite J Simis M. Evidence-based guidelines and secondary meta-analysis for the use of transcranial direct current stimulation (tDCS) in neurological and psychiatric disorders. Int J Neuropsychopharmacol. (2020) 24:256–313. 10.1093/ijnp/pyaa051 PMC8059493 32710772

[6] Kumar P. Hemiplegic shoulder pain in people with stroke: present and the future. Pain Manag. (2019) 9:107–10. 10.2217/pmt-2018-0075 30681020

[7] Roe Y Soberg HL Bautz-Holter E Ostensjo S. A systematic review of measures of shoulder pain and functioning using the International classification of functioning, disability and health (ICF). BMC Musculoskelet Disord. (2013) 14:73. 10.1186/1471-2474-14-73 23445557 PMC3668165

[8] Adey-Wakeling Z Arima H Crotty M Leyden J Kleinig T Anderson CS. Incidence and associations of hemiplegic shoulder pain poststroke: prospective population-based study. Arch Phys Med Rehab. (2015) 96:241–7.e1. 10.1016/j.apmr.2014.09.007 25264111

[9] Latremoliere A Woolf CJ. Central sensitization: a generator of pain hypersensitivity by central neural plasticity. J Pain. (2009) 10:895–926. 10.1016/j.jpain.2009.06.012 19712899 PMC2750819

[10] Lotze M Grodd W Birbaumer N Erb M Huse E Flor H. Does use of a myoelectric prosthesis prevent cortical reorganization and phantom limb pain? Nat Neurosci. (1999) 2:501–2. 10.1038/9145 10448212

[11] Parker RS Lewis GN Rice DA McNair PJ. Is motor cortical excitability altered in people with chronic pain? A systematic review and meta-analysis. Brain Stimul. (2016) 9:488–500. 10.1016/j.brs.2016.03.020 27133804

[12] Boggio PS Zaghi S Lopes M &Fregni F. Modulatory effects of anodal transcranial direct current stimulation on perception and pain thresholds in healthy volunteers. Eur J Neurol. (2008) 15:1124–30. 10.1111/j.1468-1331.2008.02270.x 18717717

[13] Bolognini N Vallar G Casati C Latif LA El-Nazer R Williams J. Neurophysiological and behavioral effects of tDCS combined with constraint-induced movement therapy in poststroke patients. Neurorehabil Neural Repair. (2011) 25:819–29. 10.1177/1545968311411056 21803933

[14] Edwards DJ Cortes M Rykman-Peltz A Chang J Elder J Gary T. Clinical improvement with intensive robot-assisted arm training in chronic stroke is unchanged by supplementary tDCS. Restorative Neurol Neurosci. (2019) 37:167–80. 10.3233/RNN-180869 30932903

[15] Turrigiano GG. Homeostatic plasticity in neuronal networks: the more things change, the more they stay the same. Trends Neurosci. (1999) 22:221–7. 10.1016/S0166-2236(98)01341-1 10322495

[16] Zimerman M Heise KF Hoppe J Cohen LG Gerloff C Hummel FC. Modulation of training by single-session transcranial direct current stimulation to the intact motor cortex enhances motor skill acquisition of the paretic hand. Stroke. (2012) 43:2185–91. 10.1161/STROKEAHA.111.645382 22618381 PMC4879963

[17] International Association for the Study of Pain (IASP). Part III: Pain Terms: A Current List With Definitions and Notes on Usage. Class. Chronic Pain. (2012). Available online at: http://www.iasp-Pain.org/PublicationsNews/Content.aspx?ItemNumber=1673&navItemNumber=677 (accessed October 22, 2018).

[18] Bertolucci PH Brucki SM Campacci SR &Juliano Y. O Mini-Exame do Estado Mental em uma população geral. Impacto da escolaridade [The Mini-Mental State Examination in a general population: impact of educational status]. Arq neuropsiquiatr. (1994) 52:1–7. 10.1590/S0004-282X1994000100001 8002795

[19] Nitsche MA Cohen LG Wassermann EM Priori A Lang N Antal A. Transcranial direct current stimulation: State of the art 2008. Brain Stimul. (2008) 1:206–23. 10.1016/j.brs.2008.06.004 20633386

[20] Bohannon RW Smith MB. Interrater reliability of a modified Ashworth scale of muscle spasticity. Phys Ther. (1987) 67:206–7. 10.1093/ptj/67.2.206 3809245

[21] Adams HP Jr Davis PH Leira EC Chang KC Bendixen BH Clarke WR. Baseline NIH stroke scale score strongly predicts outcome after stroke: a report of the Trial of Org 10172 in Acute Stroke Treatment (TOAST). Neurology. (1999) 53:126–31. 10.1212/WNL.53.1.126 10408548

[22] Bennett JA Riegel B Bittner V Nichols J. Validity and reliability of the NYHA classes for measuring research outcomes in patients with cardiac disease. Heart Lung. (2002) 31:262–70. 10.1067/mhl.2002.124554 12122390

[23] de Souza JA Corrêa JCF Agnol LD Dos Santos FR Gomes MRP Corrêa FI. Effects of transcranial direct current stimulation on the rehabilitation of painful shoulder following a stroke: protocol for a randomized, controlled, double-blind, clinical trial. Trials. (2019) 20:165. 10.1186/s13063-019-3266-y 30876431 PMC6419802

[24] Bijur PE Silver W Gallagher EJ. Reliability of the visual analog scale for measurement of acute pain. Acad Emerg Med. (2001) 8:1153–7. 10.1111/j.1553-2712.2001.tb01132.x 11733293

[25] Martins J Napoles BV Hoffman CB Oliveira AS. The Brazilian version of Shoulder Pain and Disability Index: translation, cultural adaptation and reliability. Rev Bras Fisioter. (2010) 14:527–36. 10.1590/S1413-35552010000600012 21340248

[26] Orfale AG Araújo PM Ferraz MB Natour J. Translation into Brazilian Portuguese, cultural adaptation and evaluation of the reliability of the disabilities of the arm, shoulder and hand questionnaire. Braz J Med Biol Res. (2005) 38:293–302. 10.1590/S0100-879X2005000200018 15785841

[27] Maki T Quagliato EMAB Cacho EWA Paz LPS Nascimento NH Inoue MMEA. Estudo da confiabilidade da aplicação da escala de Fugl-Meyer no Brasil. Rev Bras Fisioter. (2006) 10:177–83. 10.1590/S1413-35552006000200007

[28] Michaelsen SM Rocha AS Knabben RJ Rodrigues LP Fernandes CGC. Tradução, adaptação e confiabilidade interexaminadores do manual de administração da escala de Fugl-Meyer. Braz J Phys Ther. (2011) 15:80–8. 10.1590/S1413-35552011000100013

[29] Marques AP. Manual de Goniometria. São Paulo: Editora Manole (2003).

[30] Figueiredo I Sampaio RF Mancini MC Silva FCM Souza MAP. Teste de força de preensão utilizando o dinamômetro Jamar. Acta. Fisiatri. (2007) 14:104–10. 10.5935/0104-7795.20070002

[31] Lima RCM Teixeira-Salmela LF Magalhães LC Gomes-Neto M. Propriedades psicométricas da versão brasileira da escala de qualidade de vida específica para acidente vascular encefálico: aplicação do modelo Rasch. Bra J Physl Ther. (2008) 12:149–56. 10.1590/S1413-35552008000200012

[32] Gorenstein C Andrade L. Inventário de depressão de Beck: propriedades psicométricas da versão em português. Rev Psiquiatr Clín. (1998) 25:245–50.

[33] Gomes-Oliveira MH Gorenstein C Lotufo Neto F Andrade LH Wang YP. Validation of the Brazilian Portuguese version of the beck depression inventory-II in a community sample. Braz J Psychiatry. (2012) 34:389–94. 10.1016/j.rbp.2012.03.005 23429809

[34] Bertolazi AN Fagondes SC Hoff LS Dartora EG Miozzo IC de Barba ME. Validation of the Brazilian Portuguese version of the Pittsburgh sleep quality index. Sleep Med. (2011) 12:70–5. 10.1016/j.sleep.2010.04.020 21145786

[35] Fregni F Boggio PS Lima MC Ferreira MJ Wagner T Rigonatti SP. A sham-controlled, phase II trial of transcranial direct current stimulation for the treatment of central pain in traumatic spinal cord injury. Pain. (2006) 122:197–209. 10.1016/j.pain.2006.02.023 16564618

[36] Bae SH Kim GD Kim KY. Analgesic effect of transcranial direct current stimulation on central post-stroke pain. Tohoku J Exp Med. (2014) 234:189–95. 10.1620/tjem.234.189 25341455

[37] Lefaucheur JP Antal A Ahdab R Ciampi de Andrade D Fregni F Khedr EM. The use of repetitive transcranial magnetic stimulation (rTMS) and transcranial direct current stimulation (tDCS) to relieve pain. Brain Stimul. (2008) 1:337–44. 10.1016/j.brs.2008.07.003 20633392

[38] Nitsche MA Doemkes S Karaköse T Antal A Liebetanz D Lang N. Shaping the effects of transcranial direct current stimulation of the human motor cortex. J Neurophysiol. (2007) 97:3109–17. 10.1152/jn.01312.2006 17251360

[39] Xu J Fregni F Brody AL Rahman AS. Transcranial direct current stimulation reduces negative affect but not cigarette craving in overnight abstinent smokers. Front Psychiatry. (2013) 4:112. 10.3389/fpsyt.2013.00112 24065930 PMC3778370

[40] Belley AF Mercier C Bastien M Léonard G Gaudreault N Roy JS. Anodal transcranial direct-current stimulation to enhance rehabilitation in individuals with rotator cuff tendinopathy: a triple-blind randomized controlled trial. J Orthop Sports Phys Ther. (2018) 48:541–51. 10.2519/jospt.2018.7871 29747540

[41] Priori A Berardelli A Rona S Accornero N Manfredi M. Polarization of the human motor cortex through the scalp. Neuroreport. (1998) 9:2257–60. 10.1097/00001756-199807130-00020 9694210

[42] Harvey RL Edwards D Dunning K Fregni F Stein J Laine J. Randomized sham-controlled trial of navigated repetitive transcranial magnetic stimulation for motor recovery in stroke. Stroke. (2018) 49:2138–46. 10.1161/STROKEAHA.117.020607 30354990

[43] Antal A Terney D Kühnl S Paulus W. Anodal transcranial direct current stimulation of the motor cortex ameliorates chronic pain and reduces short intracortical inhibition. J Pain Symptom Manage. (2010) 39:890–903. 10.1016/j.jpainsymman.2009.09.023 20471549

[44] Thibaut A Carvalho S Morse LR Zafonte R Fregni F. Delayed pain decrease following M1 tDCS in spinal cord injury: A randomized controlled clinical trial. Neurosci Lett. (2017) 658:19–26. 10.1016/j.neulet.2017.08.024 28822837

[45] Massé-Alarie H Beaulieu LD Preuss R Schneider C. The side of chronic low back pain matters: evidence from the primary motor cortex excitability and the postural adjustments of multifidi muscles. Exp Brain Res. (2017) 235:647–59. 10.1007/s00221-016-4834-y 27847987

[46] Massé-Alarie H Schneider C. Revisiting the corticomotor plasticity in low back pain: challenges and perspectives. Healthcare (Basel). (2016) 4:67. 10.3390/healthcare4030067 27618123 PMC5041068

[47] Baliki MN Chialvo DR Geha PY Levy RM Harden RN Parrish TB. Chronic pain and the emotional brain: specific brain activity associated with spontaneous fluctuations of intensity of chronic back pain. J Neurosci. (2006) 26:12165–73. 10.1523/JNEUROSCI.3576-06.2006 17122041 PMC4177069

[48] Baliki MN Schnitzer TJ Bauer WR Apkarian AV. Brain morphological signatures for chronic pain. PLoS ONE. (2011) 6:e26010. 10.1371/journal.pone.0026010 22022493 PMC3192794

[49] Flor H. Maladaptive plasticity, memory for pain and phantom limb pain: review and suggestions for new therapies. Expert Rev Neurother. (2008) 8:809–18. 10.1586/14737175.8.5.809 18457537

[50] Seifert F Maihöfner C. Central mechanisms of experimental and chronic neuropathic pain: findings from functional imaging studies. Cell Mol Life Sci. (2009) 66:375–90. 10.1007/s00018-008-8428-0 18791842 PMC11131450

[51] Maarrawi J Peyron R Mertens P Costes N Magnin M Sindou M. Motor cortex stimulation for pain control induces changes in the endogenous opioid system. Neurology. (2007) 69:827–34. 10.1212/01.wnl.0000269783.86997.37 17724284

[52] França NR Toniolo EF Franciosi AC Alves AS de Andrade DC Fonoff ET. Antinociception induced by motor cortex stimulation: somatotopy of behavioral response and profile of neuronal activation. Behav Brain Res. (2013) 250:211–21. 10.1016/j.bbr.2013.05.019 23692698

[53] Fonoff ET Dale CS Pagano RL Paccola CC Ballester G Teixeira MJ. Antinociception induced by epidural motor cortex stimulation in naive conscious rats is mediated by the opioid system. Behav Brain Res. (2009) 196:63–70. 10.1016/j.bbr.2008.07.027 18718490

[54] Pagano RL Assis DV Clara JA Alves AS Dale CS Teixeira MJ. Transdural motor cortex stimulation reverses neuropathic pain in rats: a profile of neuronal activation. Eur J Pain. (2011) 15:268.e1-14. 10.1016/j.ejpain.2010.08.003 20817578

[55] Peyron R Faillenot I Mertens P Laurent B Garcia-Larrea L. Motor cortex stimulation in neuropathic pain. Correlations between analgesic effect and hemodynamic changes in the brain. A PET study. Neuroimage. (2007) 34:310–21. 10.1016/j.neuroimage.2006.08.037 17055297

[56] Viisanen H Pertovaara A. Roles of the rostroventromedial medulla and the spinal 5-HT(1A) receptor in descending antinociception induced by motor cortex stimulation in the neuropathic rat. Neurosci Lett. (2010) 476:133–7. 10.1016/j.neulet.2010.04.014 20398735

[57] Carlino E Piedimonte A Benedetti F. Nature of the placebo and nocebo effect in relation to functional neurologic disorders. Handb Clin Neurol. (2016) 139:597–606. 10.1016/B978-0-12-801772-2.00048-5 27719874

[58] Rabipour S Wu AD Davidson PS Iacoboni M. Expectations may influence the effects of transcranial direct current stimulation. Neuropsychologia. (2018) 119:524–34. 10.1016/j.neuropsychologia.2018.09.005 30227147

[59] Schambra HM Bikson M Wager TD DosSantos MF DaSilva AF. It's all in your head: reinforcing the placebo response with tDCS. Brain Stimul. (2014) 7:623–4. 10.1016/j.brs.2014.04.002 24810955 PMC4108558

[60] Lee S Walker JR Jakul L Sexton K. Does elimination of placebo responders in a placebo run-in increase the treatment effect in randomized clinical trials? A meta-analytic evaluation. Depress Anxiety. (2004) 19:10–9. 10.1002/da.10134 14978780

